# Diversity of SARS-CoV-2 isolates driven by pressure and health index

**DOI:** 10.1017/S0950268821000248

**Published:** 2021-02-01

**Authors:** R. K. Sanayaima Singh, Md. Zubbair Malik, R. K. Brojen Singh

**Affiliations:** 1 School of Computer and Systems Sciences, Jawaharlal Nehru University, New Delhi, 110067, India; 2 School of Computational and Integrative Sciences, Jawaharlal Nehru University, New Delhi, 110067, India

**Keywords:** Fractal dimension, health index, Hurst exponent, multifractal approach, pressure, SARS-CoV-2

## Abstract

One of the main concerns about the fast spreading coronavirus disease 2019 (Covid-19) pandemic is how to intervene. We analysed severe acute respiratory syndrome-coronavirus-2 (SARS-CoV-2) isolates data using the multifractal approach and found a rich in viral genome diversity, which could be one of the root causes of the fast Covid-19 pandemic and is strongly affected by pressure and health index of the hosts inhabited regions. The calculated mutation rate (*m*_r_) is observed to be maximum at a particular pressure, beyond which m_r_ maintains diversity. Hurst exponent and fractal dimension are found to be optimal at a critical pressure (*P*m), whereas, for *P* > *P*m and *P* < *P*m, we found rich genome diversity relating to complicated genome organisation and virulence of the virus. The values of these complexity measurement parameters are found to be increased linearly with health index values.

The coronavirus disease 2019 (Covid-19) pandemic is a high-risk infectious type of pneumonia [1], whose epicentre was Wuhan, Hubei Province, China [1, 2] and, affected across the world, causing a global public health emergency [3]. The pandemic is caused by severe acute respiratory syndrome-coronavirus-2 (*SARS-CoV-2*), which is a member of the *β*-CoV genus [4]. The genome of this virus is + ssRNS (single-stranded positive-sense RNA virus) [4, 5], having approximately 30 kb genome size [6] with a size 100–160 nm when it is in the compact form [5, 7, 8]. However, there is no acceptable standard theory of why SARS-CoV-2 is highly infectious to human cells and its fast human-to-human transmission capabilities. One possible hypothesis in this direction could be that the variability of viral mutations may cause the virulence nature as mutations are generally considered the basic units of species evolution [9]. These mutations are sometimes harmful and sometimes beneficial [10], but play an important role in genetic diversity via natural selection [11]. The mutation rates of RNA viruses are generally high (10^−4^−10^−6^) and prone to mutations during virus−host interaction [10]. The high mutation rate enhances the degree of viral genome replication kinetics [12, 13] and intensifies the virus's virulence and coevolution with the host [9]. It helps the viral adaptability to the host [12]. This high mutation rate of the viral genome may lead to an increase in their virulence, causing rich diversity, which could be one of the pandemic's main causes. In this letter, we report the study of the diversity in the *SARS-CoV-2* virus genome induced by pressure (quantified by sea level) and regions' health index across the world.

We have mined publicly available complete genomic data of various SARS-CoV-2 isolates (42 isolates in total) from seven countries [14]. We then calculated mutation rates (mutation per unit nucleotide) of all mined SARS-CoV-2 virus isolates. The mutation rate (*m*_r_) of an isolate can be defined by, *m*_r_ = *p*_r_/*G*_r_, where, *p*_r_ and *G*_r_ are the point mutations and the total number of nucleotides of rth isolate, respectively. Taking Wuhan seafood market isolate as the reference genome, we calculated the mutation rates (mutation per unit nucleotide) of the mined isolates as a function of height above sea level (in metres) and health index of the place from where the viral isolates were extracted (Fig. 1). The height above sea level or altitude (Λ) and dependent atmospheric pressure (*P*[Λ]) can be related to Λ by using (d*P*[Λ]/dΛ) =   − d*g* at hydrostatic equilibrium, and ideal gas equation, *P* =  (*d*/*N*)*RT* where *d* is the air density, *g* is the acceleration due to gravity, *N* is the air molar mass, *R* is the universal gas constant and *T* is the standard temperature [15], and is given by, 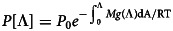, where *P*_0_ is the atmospheric pressure at sea level. The function *g*(Λ) at any height Λ can be calculated by *g*(Λ) = (*g*/*r*^2^)(*r* + Λ)^2^, where *r* is the radius of the earth. Considering a linear change in *T* (*T* = *T*_0_ + *L*Λ), where *T*_0_ is the sea level standard temperature, and *L* is the temperature lapse state of the air, we can get1
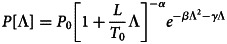
where *α* = (*Mg*/*r*^2^*LRT*)[*r*^2^ − (*T*_0_/*L*)(2*r* −  (*T*_0_/*L*))], *β* = (*Mg*/2*r*^2^*LRT*) and *γ* = (*Mg*/*r*^2^*LRT*)[2*r* −  (*T*_0_/*L*)]. Equation (1) clearly shows that pressure *P*[Λ] decreases quite fast as Λ increases. The mutation rate of the isolates (*m_r_*) is found to be relatively high (*m_r_* ∈ (10^−4^−10^−5^)), and increases as Λ increases (decrease in pressure *P*[Λ]) upto a maximum value (at around Λ ~ 1.7 × 10^2^*m*), and then decreases as Λ increases (Fig. 1, upper panel). This implies that the diversity of the viral genomes is quite dependent on host cells adapted to a certain pressure *P*. Further, the dependence of *m_r_* on *P*[Λ] indicates that viral mutation rates can evolve with pressure, adapted with the host cells, and could optimise the rate at a selective pressure. This could be because virus−host cell interaction mechanisms, which alter viral replication kinetics, proofreading, etc., might depend on the adapted pressure. The pressure-dependent richness in viral mutation diversity indicates that there is a high chance of multiple virus−host-dependent processes [16] and could lead to more infectious nature of the SARS-CoV-2 virus.

**Fig. 1.**
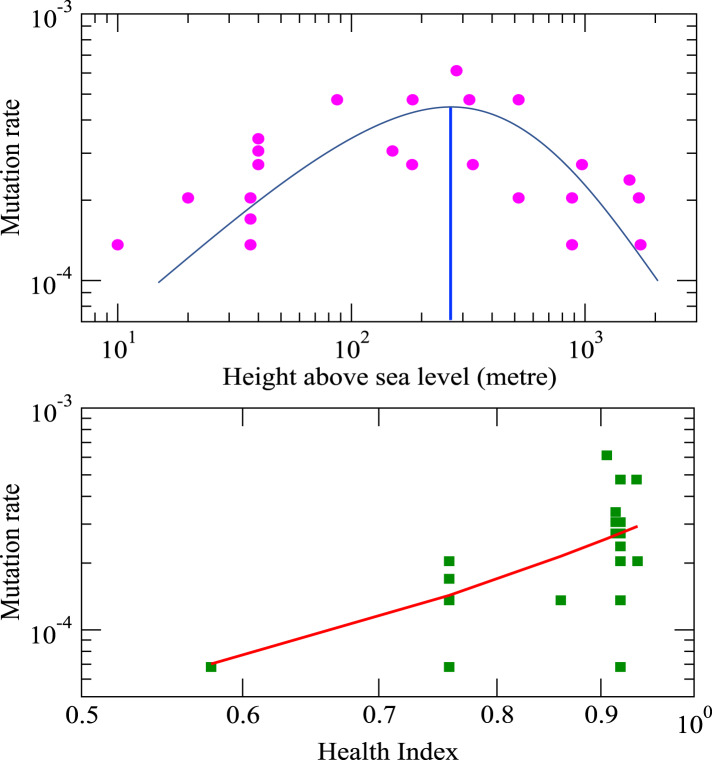
Mutation rates of SARS-CoV-2 isolates induced by height and health index: The upper panel is the plot of mutation rate as a function of height above sea level (pink filled circles are calculated data points and curve line is the fitted curve on the data points). Lower panel is the plot of mutation rate with respect to health index, where, filled squares are calculated values and red line is the fitted curve on the data points.

The health index of a place characterises the overall health of the population at that particular place. The calculated mutation rate is strongly dependent on the health index (Fig. 1, lower panel). If Γ indicates health index parameter, then the relationship of *m_r_* with Γ can be obtained by fitting the data and is found to obey, *m_r_* ~ *ae^b^*^Γ^, where *a* = 1.3713 and *b* = 0.3681 are constants. The Pearson correlation coefficient value of the fitted function to the data points is *r*^2^ = 0.8313.

Next, we study the impact of Λ and Γ on the genome complexity of the SARS-CoV-2 isolates by calculating the complexity measurement parameters, Hurst exponent (*H*), and generalised fractal dimension (*D*) [17, 18]. The biological and cellular processes of macro and micro-organisms are significantly changed by variations in atmospheric pressure and temperature characterised by the altitude parameter Λ [19]. These variations could lead to a change in genetic expression [20–22], affecting mutation frequency [23]. From the data analysis, it has also been reported that atmospheric pressure can induce a change in mutational frequency of SARS-CoV-2 virus [23, 24], and temperature can trigger SARS-CoV-2 infection rate [25]. Moreover, variation in this parameter Λ observed at various geographical locations across the world provides different environmental impacts with diversity in ecosystems causing variation in the infection rate of Covid-19 [26, 27]. Hence, this change in Λ could induce perturbation to the complicated dynamics of human−SARS-CoV-2 virus interaction, which may cause the mutational profile of this virus, causing a change in genome organisation and regulation. The procedure of calculating the effect of Λ on any SARS-CoV-2 genome at any *P* and Γ is as follows. First, we converted the symbolic genome sequence to time series like *DNA walk* by taking purine (*A* or *G*) as step-up (+1) and pyrimidine as step down (−1) [28]. By construction, *DNA walk* is a map of the genome's cumulative sum, which carries the complex information of the genome. Then *DNA walks* of 42 SARS-CoV-2 isolates collected from the patients' data are calculated as a function of Λ and Γ. Now consider a DNA walk of length *N* of a particular isolate. From this DNA walk, we explain the procedure briefly for calculating various multifractal parameters discussed as follows [18]. First, we calculated the profile function, 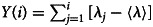 by constructing a series of length segments *λ*_*j*_'s from the DNA walk of length *N*, with *j* = 1, 2, 3, …, *N*, such that, *λ*_*j*_ = 0 is considered to be insignificant. Then, this function *Y*(*i*) is divided into *N*_*x*_ = *int*(*N*/*x*) equal non-overlapping segments of length *x*. To incorporate the end effects of *Y*(*i*), 2*N*_*x*_ segments of length *x* are considered by taking into account opposite end repetition in the simulation. The local trend of fluctuation of each 2*N*_*x*_ was estimated from the variance, which was calculated by the least-squares fitting procedure for each segment series. Averaging over all the calculated local fluctuations of all 2*N*_*x*_ segments, the *q* (order parameter) dependent *fluctuation function F_q_*(*x*) for the considered virus isolate, and found to follow fractal nature *F_q_*(*x*) ~ *x^Hq^*, where, *H_q_* is the *q* order Hurst exponent [28, 29]. Since *F_q_*(*x*) is both *q*-dependent (inter-event dependent [16]) and *x* - dependent (local domains), each genome exhibits multifractal property [17, 18, 30]. Then, *H* of each genome is obtained by *H* = <*H_q_*>. The calculated values of *H* of all SARS-CoV-2 isolates are found to be quite sensitive to *q*, indicating rich heterogeneous structures in the genomes. Further, it is also found that 1 > *H*>0.5 (Fig. 2, upper two panels), characterising genomic signal in each isolate due to long-range positive correlations in their topology [31]. This could be the evidence of strong self-organisation in each virus isolate [17, 32]. It is also found that *H* decreases with Λ till it attains minimum value *H*_min_ = 0.9151 at Λ ~ 887*m*, and then increases with Λ following, *H* ~ *u*Λ^2^ + *v*Λ + *w*, where, fitted parameter values are *u* = 10^−8^, *v* = − 2.4 × 10^−6^ and *w* = 0.916 with Pearson's correlation coefficient value *r*^2^ = 0.3135. From equation (1), taking (*L*/*T*_0_) < 1, one can approximate the factor 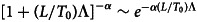, such that after simplification, we have, 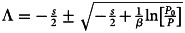, where *s* = (1/*β*)(*r* + *α*(*L*/*T*_0_)). Further, for positive Λ > 0, it can be shown that *P*_0_ > *P*. Now, putting the expression for Λ to the equation of *H*, and after simplification, we arrive at2
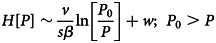

**Fig. 2.**
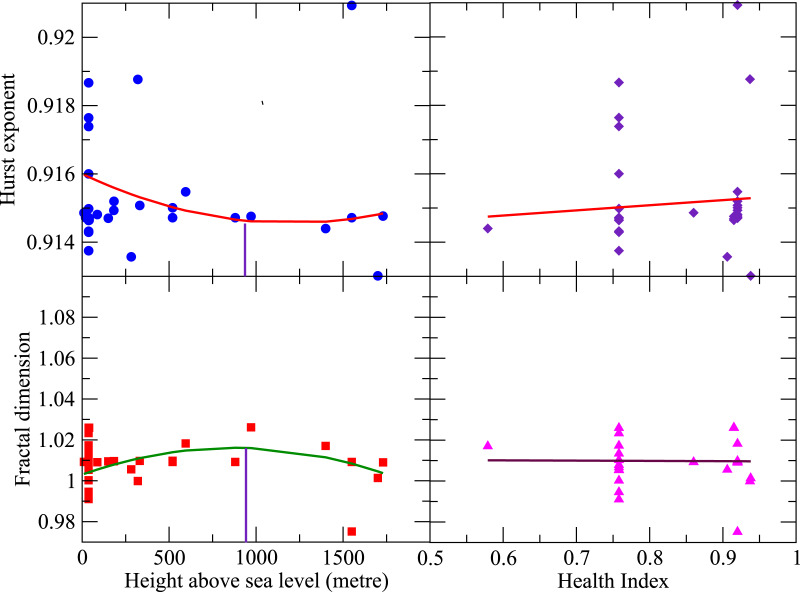
Hurst exponent and fractal dimension diversified by height above sea level and health index: Panels in the first column are the change of Hurst exponent and fractal dimension with respect to height above sea level. Panels in the second column are the plots of Hurst exponent and fractal dimension as a function of health index.

Now, Figure 2, upper left panel and equation (2) show that the virus isolates in hosts at pressure regions *P* > *P_m_* and *P* < *P_m_* (*P_m_* → Λ = 887*m*) exhibit complicated divergence indicating more virulence attempting to establish long-range correlation within the genome during the virus−host interaction. However, the virus may likely cause minimal harm to the host adapted to the regions at and around *P* → *P_m_*. The pressure variation driven by the change in altitude Λ can perturb gene expression and physiological changes of the organisms and micro-organisms [20–22]. It may cause variation of mutational frequency in the SARS-CoV-2 virus genome [23]. Our data analysis indicates that initially, the mutation rate increases as height above the sea level (Λ) increases till Λ_m_ i.e. as pressure decreases till *P*_m_ (Fig. 1, upper panel), where the mutation rate is maximum, causing an increase in viral virulence [24]. Hence it increases in infection rate as well [25]. Afterward, the mutation rate decreases as Λ increases i.e. pressure decreases, as evident from [26, 32]. This impact of pressure is always associated with the change in temperature supplementing changes in gene expressions and mutation rates, triggering the Covid-19 infection rate [25, 33]. Moreover, the change in pressure also causes variation in human lung epithelial tissue cells due to variation in respiration rate to intake oxygen, which may cause physiological changes and even various other diseases [34, 35]. Hence, even at the gene-expression level, these physiological changes may cause complications in human−SARS-CoV-2 virus interaction dynamics, driving changes in the mutation rate, leading to variation in infection rate. Now, *q*th-order generalised fractal dimension *D_q_* for each isolate can be calculated from the corresponding DNA walk by3
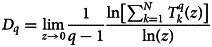
where, 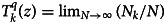 is the probability that *k*th DNA walk segment of length scale *z* will have *N_k_* observations, and obeys *T_k_^q^*(*z*) ~ *z^ν^* [29], where *ν* is Holder exponent [36]. The fractal dimension *D* can be obtained by *D* = <*D*>. Similar to *H*, *D* is also quite sensitive to *q*, indicating rich heterogeneous structure in each virus isolate [17], where local topologies might have significant functions. *D* is found to be maximum at Λ = 887*m* (Fig. 2, lower left panel) and decreases for Λ < 887*m* and Λ > 887*m*. This indicates that virus virulence is quite significant to hosts adapted at high- and low-pressure regions, and may cause the least harm to the hosts adapted at regions at and around Λ ~ Λ_0_( = 887). The degree of viral complexity measured by *H* and *D* is dependent on *health index I_H_* (Fig. 2, panels of right-hand column). The behaviour of *H* is found to be linearly dependent on *I_H_*, *H* = *εI_H_* + *δ*, where the fitted parameter values are *ε* = 0.9139, *δ* = 0.0016, and Pearson's correlation coefficient value is *r*^2^ = 0.3512. Similarly, *D* also has a similar nature as in *H*, and found that *D* depends linearly with *I_H_*, *D* = *ηI_H_* + *σ*, where, *η* = −0.001369, *σ* = 1.0109 and *r*^2^ = 0.4831. Since the virus complexity increases as *I_H_* increases, it may be the case where viral diversity might have increased in healthy hosts in order to survive in the host. We also found multiple isolates found in some countries, where, USA and Wuhan have many isolates with multiple values of fractal dimension and Hurst exponent corresponding to a particular health index assigned for a country. In general, *H* and *D* are multiple-valued functions (dependent on local trended fluctuations, which are dependent on order parameter *q*) because of multifractality of the SARS-CoV-2 viral genomes. However, here, *H* and *D* are calculated average values of each genome.

In conclusion, it is quite evident that the mutation rate and rich diversity in SARS-CoV-2 genome complexity are strongly driven by pressure levels of the hosts' inhabited regions and health index. Our results show clear exponential dependence of health index with mutation rate, which may trigger the SARS-CoV-2 virus's virulence, which may increase the infection rate. From our analysis, since higher health index people have high mutation rates causing higher infection rates, proper precautions like WHO guidelines should be strictly followed, and the immune system has to be kept strong to fight the virus infection. Our analysis of the viral isolates data show a critical pressure at which the virus causes minimal harm to the host and beyond which the viral evolution preserves rich diversity relating to virulence. The virus's complexity increases as the hosts' health index, probably for its survival and then attacks the hosts. The variation in atmospheric pressure triggers significant changes in gene expressions leading to indicative biological and physiological changes in the macro and micro-organisms [20–22]. In the Covid-19 pandemic case, there is a complicated human−SARS-CoV-2 virus interaction dynamics driven by pressure, which is associated with temperature [26]. Our data analysis showed that as with a decrease in pressure (increase in height above sea level), the mutation rate of SARS-CoV-2 virus increases until it reaches a maximum value. The virus showed maximum virulence, which may have a maximum tendency to spread in the population [24, 25, 28]. Then as pressure increases further, the mutation rate starts decreasing, indicating less virus virulence, which may cause a decrease in the infection rate in the population. Hence, we propose that these two parameters could be of high concern for analysis to intervene in the fast progressing Covid-19 pandemic.

## Data Availability

All data generated and/or analysed during the current study are available from the corresponding author on reasonable request.
